# Supplementary material for: Functional crosstalk between the cohesin loader and chromatin remodelers
Source: Nat Commun. 2022 Dec 13;13:7698. doi: 10.1038/s41467-022-35444-6 (PMC9744909; doi:10.1038/s41467-022-35444-6)
Supplement: Supplementary file 1 — Supplementary Information [file 41467_2022_35444_MOESM1_ESM.pdf]

Functional crosstalk between the cohesin loader and chromatin remodelers

Sofía Muñoz, Andrew Jones, Céline Bouchoux, Tegan Gilmore, Harshil Patel & Frank Uhlmann

Supplementary Information

	Pages
Supplementary Figures 1 – 7	2 – 8
Supplementary Table 1	9
Supplementary Table 2	10 – 11
Supplementary References	12

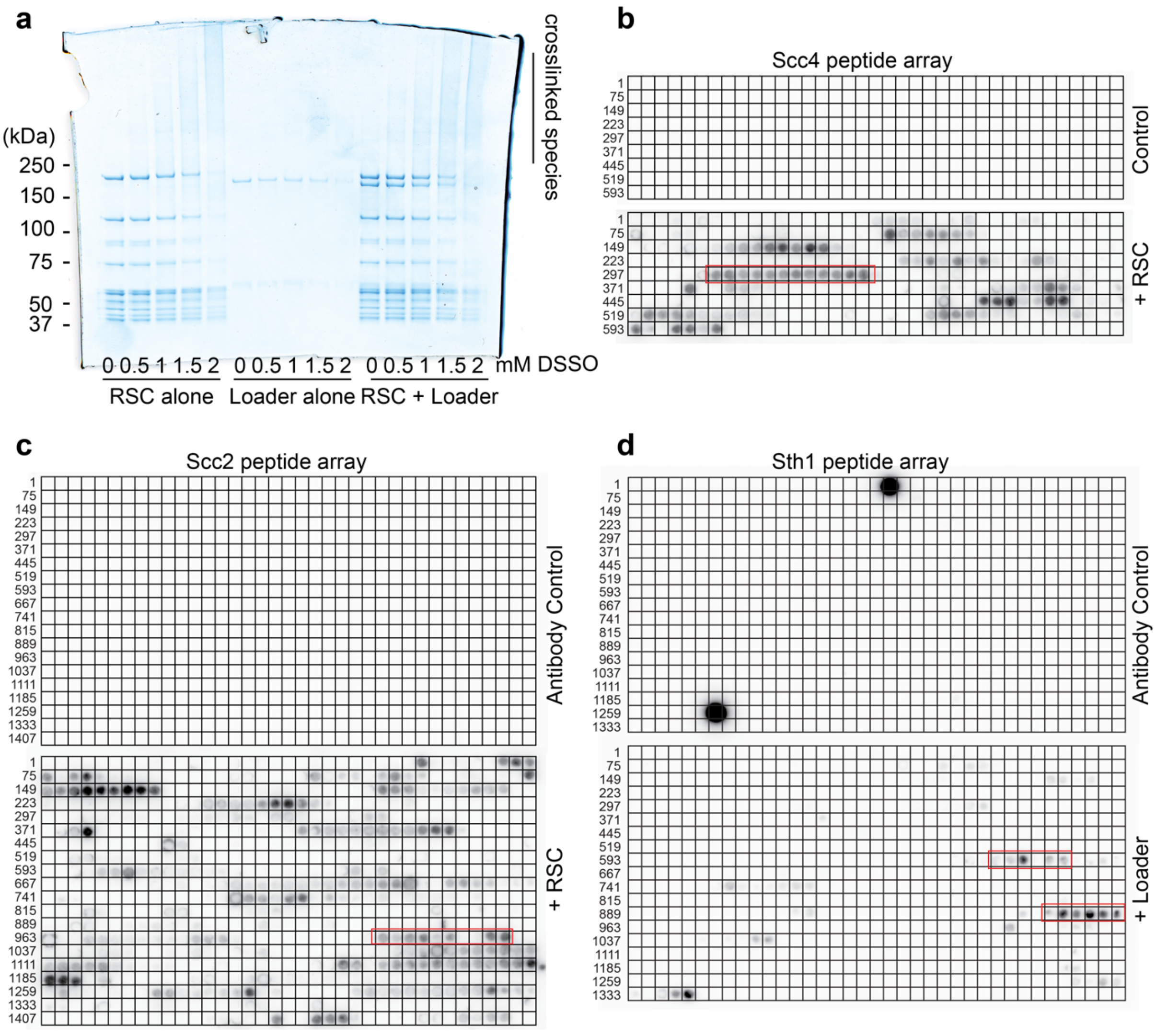

Supplementary Fig. 1 Cross-linker titration, as well as peptide array interaction analyses. a RSC, the cohesin loader, or equimolar concentrations of both, were crosslinked with the indicated concentrations of DSSO. Crosslinked species are detected as slow-migrating products during gel electrophoresis. 1 mM DSSO was chosen to prepare samples for crosslinking mass spectrometry (CLMS) analysis. This titration was conducted once, before two biological repeats of the crosslinking experiment were performed. **b, c** Identification of potential RSC binding sites using tiling Scc4 and Scc2 peptide arrays, respectively. Starting positions of the first peptide in each row are indicated. **d** Potential cohesin loader binding sites identified using an Sth1 peptide array. The red rectangles highlight peptides surrounding CLMS contacts. Source data are provided as a Source Data file.

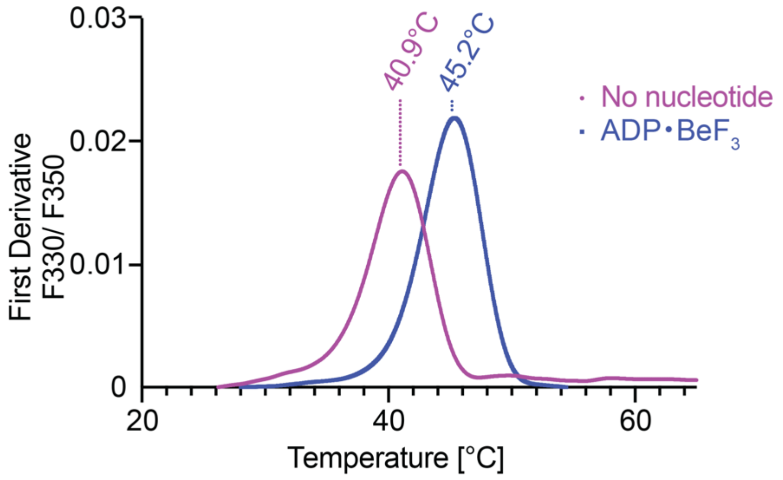

Supplementary Fig. 2 Thermal denaturation curves of the Sth1$^{365\text{-}1097}$ ATPase fragment. Melting curves of Sth1$^{365\text{-}1097}$ alone and Sth1$^{365\text{-}1097}$ in the presence of ADP・BeF_3 were recorded by nanoscale differential scanning fluorimetry, demonstrating the increased stability of the Sth1$^{365\text{-}1097}$ ATPase fragment in the presence of the non-hydrolyzable ATP analog. Source data are provided as a Source Data file.

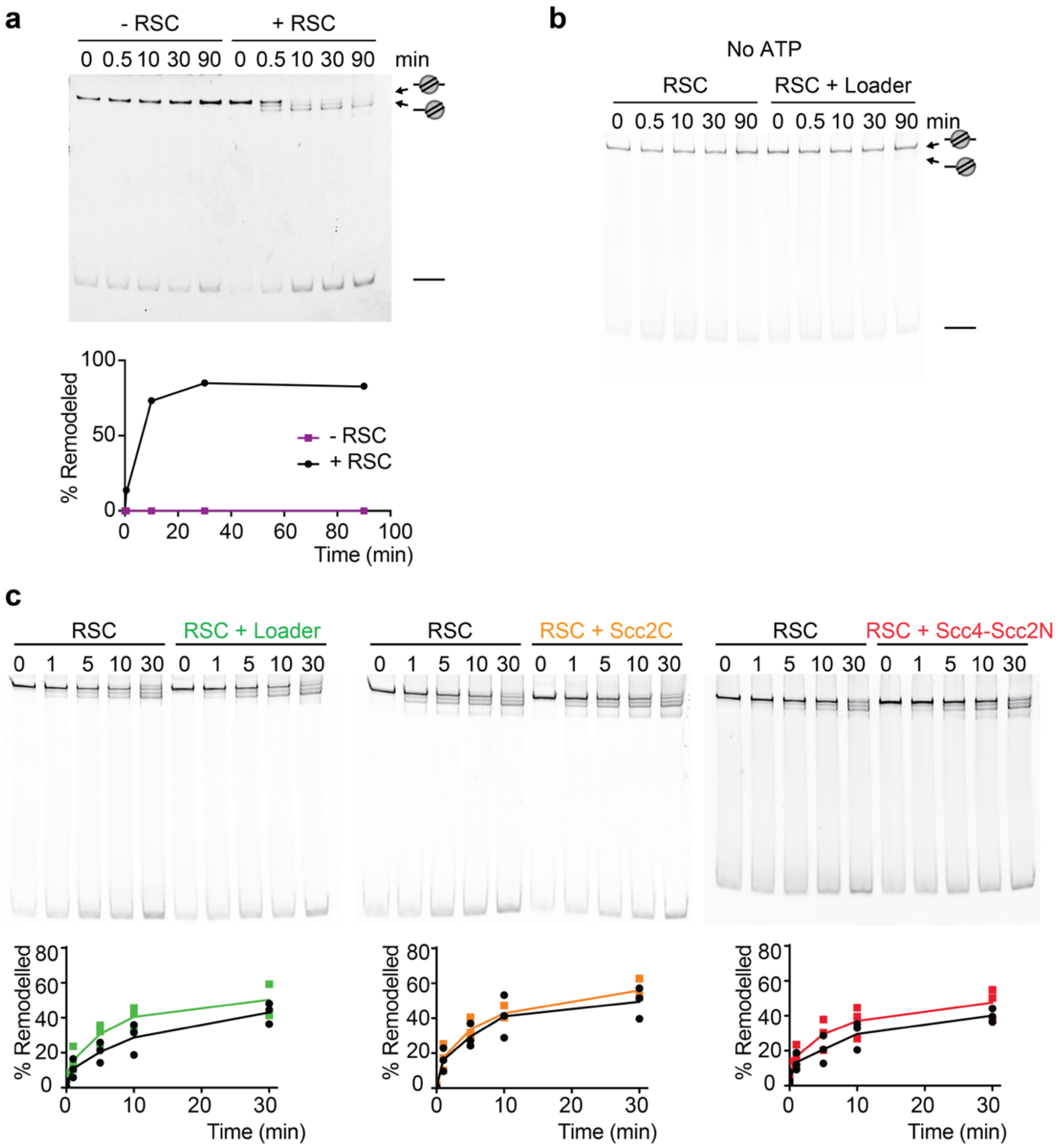

Supplementary Fig. 3 RSC nucleosome remodeling assay. a A mononucleosome substrate was incubated with or without RSC. Appearance of a faster migrating band, indicative of the remodeled product following nucleosome sliding, depended on the presence of RSC (substrate and product are marked by arrows). **b** ATP-dependence of the remodeling reaction. The assay was repeated, with or without added cohesin loader, but in the absence of ATP. The experiments shown in panels **a** and **b** were performed once, as controls for experiments shown in Figure 3. **c** Accelerated nucleosome sliding in presence of the Scc2-Scc4 cohesin loader (p = 0.0062, two-way ANOVA test), or its Scc2C or Scc4-Scc2N modules. While both modules increased the remodeling efficiency, the effect was significant only in the case of the Scc4-Scc2N module (p = 0.022, two-way ANOVA test compared to RSC). Source data are provided as a Source Data file.

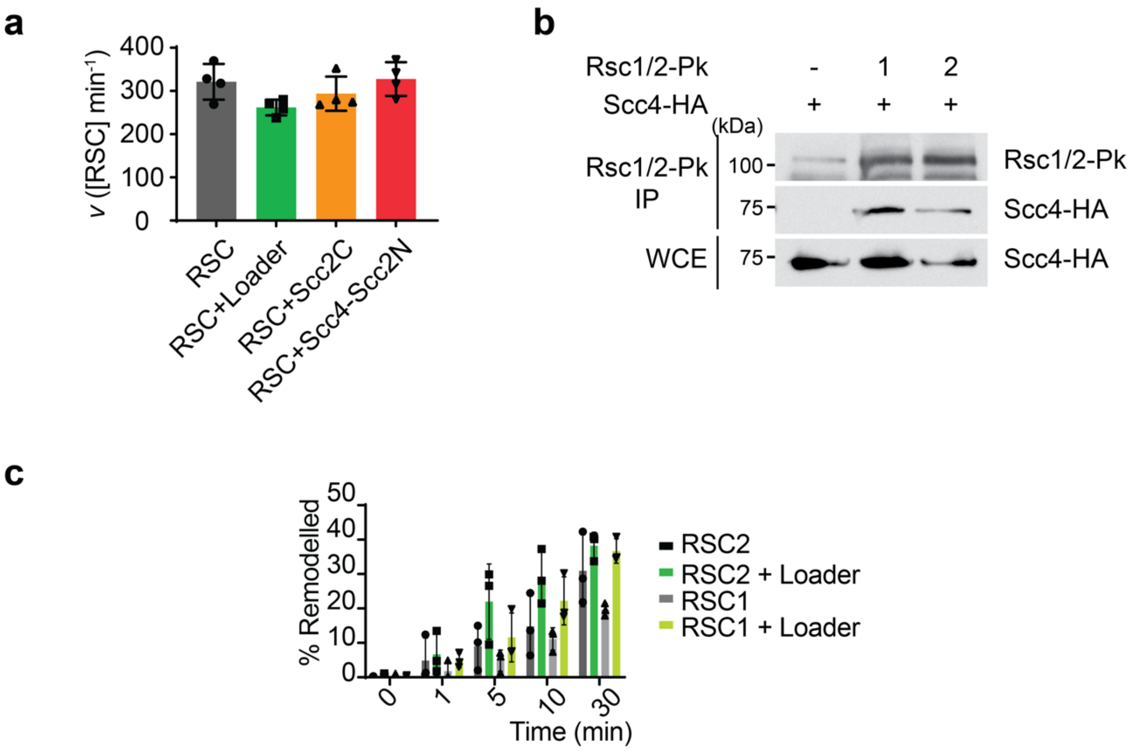

Supplementary Fig. 4 ATP hydrolysis rates and the RSC1 and RSC2 complexes. **a** ATP hydrolysis rates of RSC in the presence of a nucleosomal substrate, as well as absence or presence of the cohesin loader, Scc2C or Scc4-Scc2N. Means and SD from four biological repeat analyses are shown. No significant differences were detected by a two-sided Student's *t*-test. **b** RSC1 and RSC2 interact with the cohesin loader. Co-immunoprecipitation of the RSC1 and RSC2 complexes with the cohesin loader (detected by an HA epitope on its Scc4 subunit) were analyzed by immunoblotting. Proteins were analyzed on a 10% polyacrylamide gel, which compresses the gel mobility difference between Rsc1 and Rsc2 seen on a lower percentage gradient gel (Fig. 3c). The experiment was repeated twice with similar outcome. **c** Quantification of three biological repeats of a side-by-side comparison of chromatin remodeling by RSC1 and RSC2 in the absence or presence of the cohesin loader. Means and SD from three biological repeat analyses are shown. Source data are provided as a Source Data file.

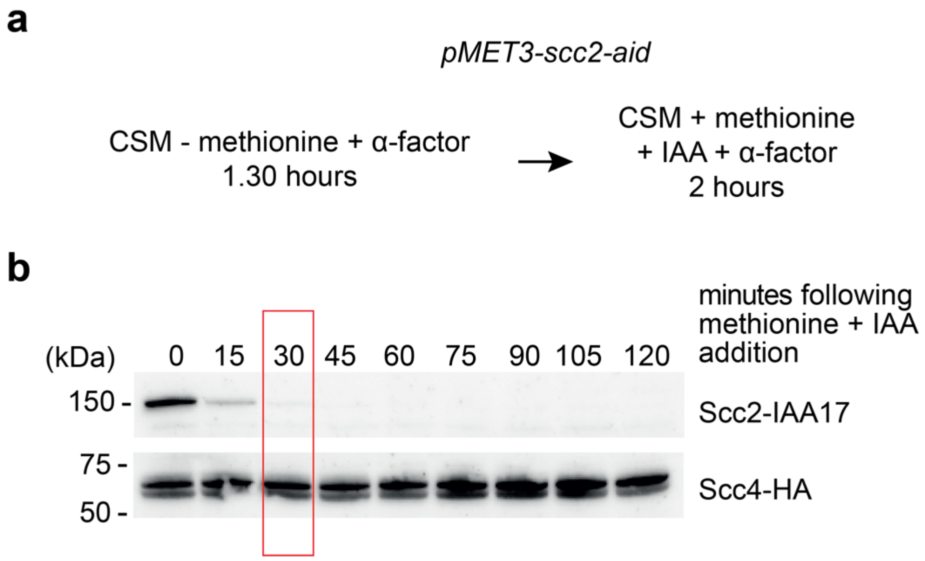

Supplementary Fig. 5 Experimental design and Scc2 depletion during the MNase-seq experiment. a Experimental design to deplete Scc2 in G1 arrested cells before nucleosome positioning analysis. **b** Time course analysis of Scc2 and Scc4 levels. Scc2 levels were detected via an aid-tag antibody, at 15-minute intervals following addition of methionine to repress Scc2 expression and of indole-3-acetic acid (IAA) to promote its degradation. The experiment was repeated twice with similar outcome. Scc4 levels, detected via an HA epitope-tag were also monitored. The red rectangle highlights the timepoint of the MNase-seq experiment shown in Figure 4.

While Scc2 was efficiently degraded following promoter shut-off and auxin addition, Scc4 remained stable. This contrasts with experiments when Scc2 is depleted while cells progress from G1 phase through S phase and into mitosis. Then Scc4 becomes unstable and its levels substantially decrease. Remaining Scc4 in the absence of Scc2, however, is unable to promote cohesin loading onto chromosomes and on its own does not interact with Sth1[1]. Source data are provided as a Source Data file.

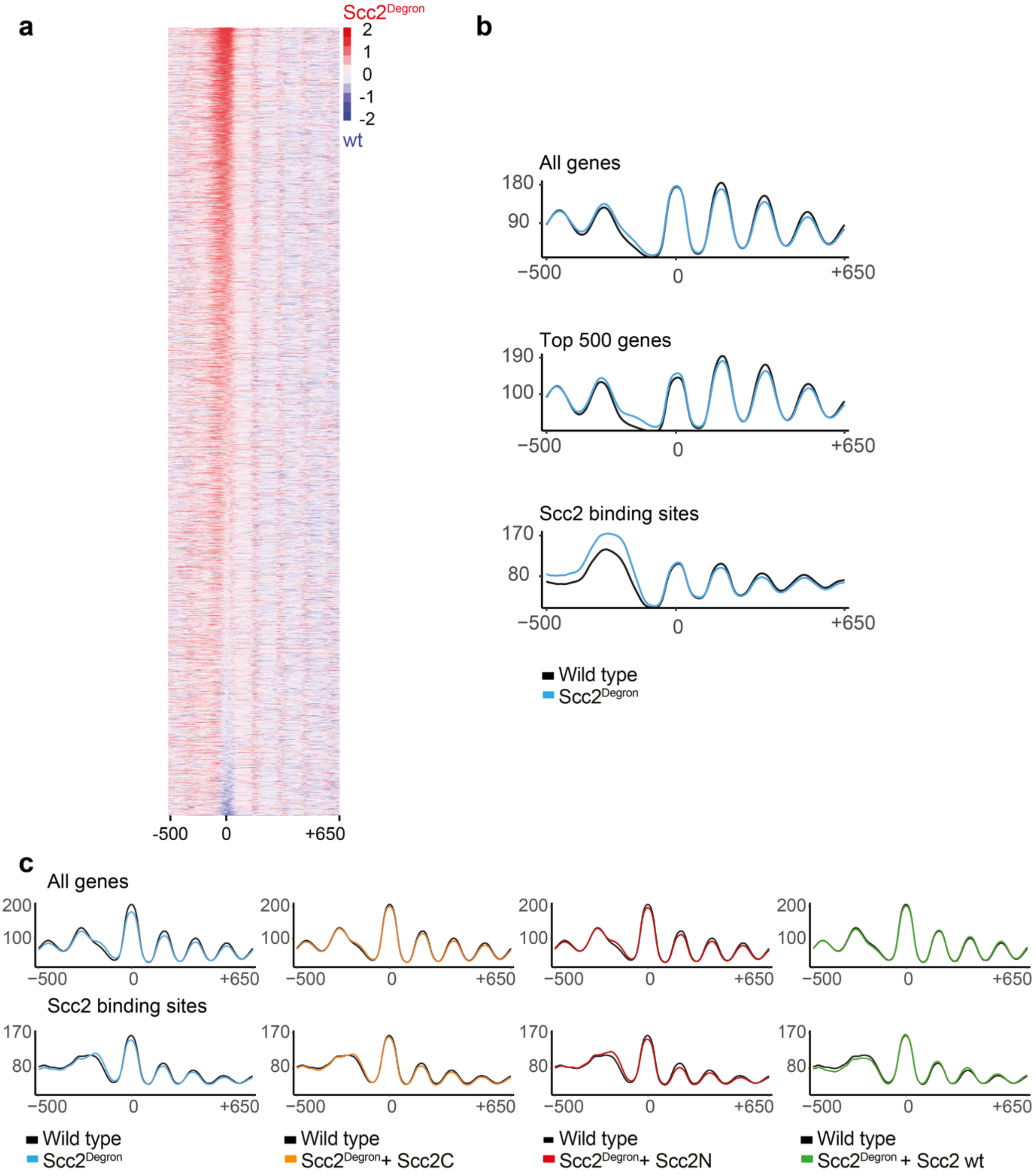

Supplementary Fig. 6 Promoter nucleosome eviction by the cohesin loader. a Differential nucleosome positioning heat map, centered on the +1 nucleosome midpoint, comparing Scc2 depletion to the wt control strain (scale, log_2). **b** Average promoter nucleosome occupancy profiles across all genes, the 500 genes that showed the greatest difference (within a -150 to -50 interval), and all those genes previously identified as cohesin loader binding sites[2]. This is a repeat of the experiment shown in Figure 4 and Supplementary Figure 6c. **c** Average nucleosome occupancy profiles from the experiment shown in Figure 4, extending to all genes or to those genes previously identified to contain cohesin loader binding sites[2].

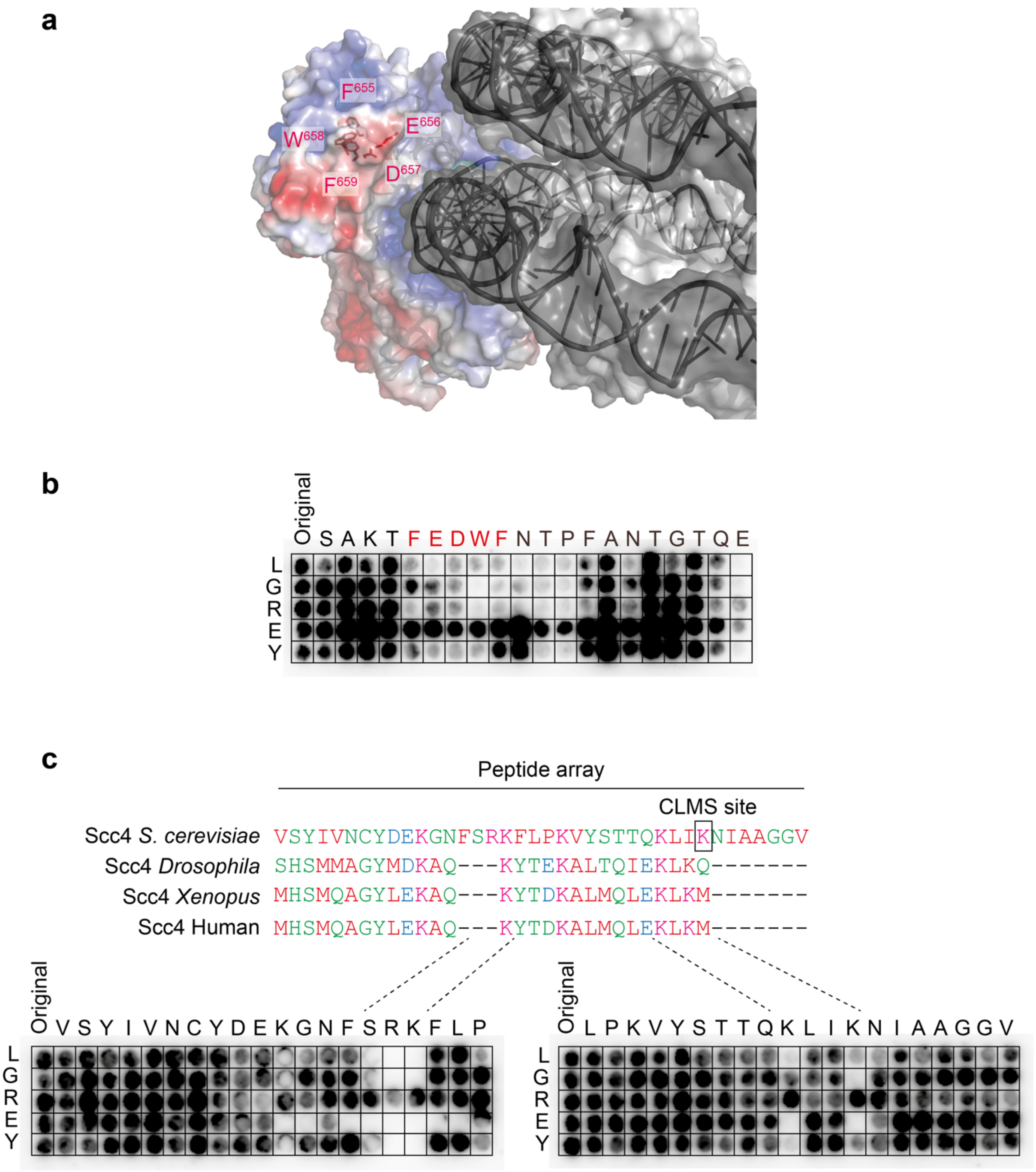

Supplementary Fig. 7 Analysis of the FEDWF motif interaction with Scc4. The conserved FEDWF motif in Sth1 ATPase module is highlighted on the cryo-EM structure of the budding yeast RSC complex (PDB: 6KW3)[3]. **b** Mutational Sth1 peptide array analysis reveals the importance of the FEDWF motif for the cohesin loader interaction. **c** Mutational peptide array analysis highlights conserved residues within Scc4 that are important for the interaction with RSC. Source data are provided as a Source Data file.

Supplementary Table 1 Plasmids used in this study

Name	Description	Purpose	Source
pSM93	pGEX-6P-2-Sth1$^{365-1097}$	Sth1$^{365-1097}$ purification	This study
pSMH439	His6-TEV-Scc2$^{1-181}$ His6-TEV-Scc4	Scc4-Scc2N purification	(Hinshaw *et al.*, 2015)[4]
pSM74	His6-TEV-Scc2$^{1-181}$ His6-TEV-Scc4-HA	HA tagged Scc4-Scc2N purification	This study
	pCDFduet.H2A-H2B	Histone purification	(Kingston *et al.*, 2011)[5]
	pETduet.H3-H4	Histone purification	(Kingston *et al.*, 2011)[5]
p1380	Vector carrying the Widom 601 sequence	Mononucleosome substrate preparation	(Lesbats *et al*, 2017)[6]
pSM83	pRS303-Scc2-HA_3	Integration of wild type Scc2-HA_3 at *HIS3* locus	This study
pSM84	pRS303-Scc2C-HA_3	Integration of wild type Scc2C-HA_3 at *HIS3* locus	This study
pSM85	pRS303-Scc2N-HA_3	Integration of wild type Scc2N-HA_3 at *HIS3* locus	This study

Supplementary Table 2 Yeast strains used in this study

Name	Genotype	Purpose	Source
Y5278	*MAT***a** *pep4Δ::HIS3 pGAL-SCC2-3C-PA$_2$::ADE2 pGAL-SCC4-HA$_3$::TRP1*	Cohesin loader purification	(Minamino *et al*, 2018)[7]
Y5542	*MAT***a** *pep4Δ::HIS3 prblΔ::LEU2 prcΔ::HISG RSC2-TAP::TRP1*	RSC purification	(Wittmeyer *et al*, 2004)[8]
Y5617	*MAT***a** *pep4Δ::HIS3 prblΔ::LEU2 prcΔ::HISG RSC2-TAP::TRP1 STH1-PK$_3$::KANMX6*	RSC with PK tagged Sth1 purification	This study
Y5560	*MAT***a** *pep4Δ::HIS3 pGAL-HA$_3$-SCC2C-3C-PA$_2$::ADE2*	Scc2C purification	(Minamino *et al*, 2018)[7]
Y6240	*MAT***a** *SCC2-HA$_3$::LEU2*	Coimmunoprecipitation cohesin loader-RSC1 or RSC2	This study
Y6238	*MAT***a** *SCC2-HA$_3$::LEU2 RSC1-PK3::TRP1*	Coimmunoprecipitation cohesin loader-RSC1 or RSC2	This study
Y6239	*MAT***a** *SCC2-HA$_3$::LEU2 RSC2-PK3::TRP1*	Coimmunoprecipitation cohesin loader-RSC1 or RSC2	This study
Y4842	*MAT***a** *SCC4-HA$_3$::LEU2*	Coimmunoprecipitation cohesin loader-RSC1 or RSC2	(Muñoz *et al*, 2019)[1]
ABN88.80	*MAT***a** *RSC1-PK3::TRP1 SCC4-HA$_3$::K.l.URA*	Coimmunoprecipitation cohesin loader-RSC1 or RSC2	This study
ABN88.81	*MAT***a** *RSC2-PK3::TRP1 SCC4-HA$_3$::K.l.URA*	Coimmunoprecipitation cohesin loader-RSC1 or RSC2	This study
Y6235	*MAT***a** *pep4Δ::HIS3 RSC1-TAP::TRP1*	RSC1 purification	This study
Y4267	*MATa pADH1-OsTir1-Myc9::ADE2*	Mnase-seq experiments	
Y4809	*MAT***a** *pADH1-OsTir1-Myc$_9$::ADE2 URA3::pMET3::SCC2-IAA17::KANMX6*	Mnase-seq experiments	This study
Y4816	*MAT***a** *pADH1-OsTir1-Myc$_9$::ADE2 URA3::pMET3::SCC2-IAA17::KANMX6 SCC4-HA3::LEU2*	Mnase-seq experiments	This study
Y6276	*MAT***a** *pADH1-OsTir1-Myc$_9$::ADE2 URA3::pMET3::SCC2-IAA17::KANMX6 SCC2-HA$_3$::HIS3*	Mnase-seq experiments	This study
Y6277	*MAT***a** *pADH1-OsTir1-Myc$_9$::ADE2 URA3::pMET3::SCC2-IAA17::KANMX6 SCC2C-HA$_3$::HIS3*	Mnase-seq experiments	This study
Y6278	*MAT***a** *pADH1-OsTir1-Myc$_9$::ADE2 URA3::pMET3::SCC2-IAA17::KANMX6 SCC2N-HA$_3$::HIS3*	Mnase-seq experiments	This study
Y4373	*MAT***a** *SCC4-PK$_9$::TRP1*	Coimmunoprecipitation cohesin loader-all remodeller families *S. cerevisiae*	(Lopez-Serra *et al*, 2014)[2]
Y4394	*MAT***a** *SCC4-PK$_9$::TRP1 STH1-PA$_2$::HIS3*	Coimmunoprecipitation cohesin loader-all remodeller families *S. cerevisiae*	This study
Y4395	*MAT***a** *SCC4-PK$_9$::TRP1 SNF2-PA$_2$::HIS3*	Coimmunoprecipitation cohesin loader-all remodeller families *S. cerevisiae*	This study
Y6073	*MAT***a** *SCC4-PK$_9$::TRP1 ISW1-PA$_2$::HIS3*	Coimmunoprecipitation cohesin loader-all remodeller families *S. cerevisiae*	This study

Y6074	*MAT***a** *SCC4-PK$_9$::TRP1 ISW2-PA$_2$::HIS3*	Coimmunoprecipitation cohesin loader-all remodeller families *S. cerevisiae*	This study
Y4465	*MAT***a** *SCC4-PK$_9$::TRP1 CHD1-PA$_2$::HIS3*	Coimmunoprecipitation cohesin loader-all remodeller families *S. cerevisiae*	This study
Y4440	*MAT***a** *SCC4-PK$_9$::TRP1 INO80-PA$_2$::HIS3*	Coimmunoprecipitation cohesin loader-all remodeller families *S. cerevisiae*	This study
Y4462	*MAT***a** *SCC4-PK$_9$::TRP1 SWR1-PA$_2$::HIS3*	Coimmunoprecipitation cohesin loader-all remodeller families *S. cerevisiae*	This study
Y5987	*h^-, Ssl3-PK3::hphMX, leu1-32, ura4-D18*	Coimmunoprecipitation cohesin loader-all remodeller families *S. pombe*	(Muñoz *et al*, 2020)[9]
Y5988	*h^-, Ssl3-PK3::hphMX, Snf21-PA::kanMX, leu1-32, ura4-D18*	Coimmunoprecipitation cohesin loader-all remodeller families *S. pombe*	(Muñoz *et al*, 2020)[9]
Y6028	*h^-, Ssl3-PK3::hphMX, Snf22-PA::kanMX, leu1-32, ura4-D18*	Coimmunoprecipitation cohesin loader-all remodeller families *S. pombe*	This study
Y6030	*h^-, Ssl3-PK3::hphMX, Hrp1-PA::kanMX, leu1-32, ura4-D18*	Coimmunoprecipitation cohesin loader-all remodeller families *S. pombe*	This study
Y6031	*h^-, Ssl3-PK3::hphMX, Hrp3-PA::kanMX, leu1-32, ura4-D18*	Coimmunoprecipitation cohesin loader-all remodeller families *S. pombe*	This study
Y6032	*h^-, Ssl3-PK3::hphMX, Mit1-PA::kanMX, leu1-32, ura4-D18*	Coimmunoprecipitation cohesin loader-all remodeller families *S. pombe*	This study
Y6029	*h^-, Ssl3-PK3::hphMX, Ino80-PA::kanMX, leu1-32, ura4-D18*	Coimmunoprecipitation cohesin loader-all remodeller families *S. pombe*	This study
Y6033	*h^-, Ssl3-PK3::hphMX, Swr1-PA::kanMX, leu1-32, ura4-D18*	Coimmunoprecipitation cohesin loader-all remodeller families *S. pombe*	This study

Supplementary References

1. Muñoz, S., Minamino, M., Casas-Delucchi, C. S., Patel, H. & Uhlmann, F. A Role for Chromatin Remodeling in Cohesin Loading onto Chromosomes. *Mol Cell* **74**, 664-673 e665 (2019).
2. Lopez-Serra, L., Kelly, G., Patel, H., Stewart, A. & Uhlmann, F. The Scc2-Scc4 complex acts in sister chromatid cohesion and transcriptional regulation by maintaining nucleosome-free regions. *Nat Genet* **46**, 1147-1151 (2014).
3. Ye, Y. *et al.* Structure of the RSC complex bound to the nucleosome. *Science* **366**, 838-843 (2019).
4. Hinshaw, S. M., Makrantoni, V., Kerr, A., Marston, A. L. & Harrison, S. C. Structural evidence for Scc4-dependent localization of cohesin loading. *Elife* **4**, e06057 (2015).
5. Kingston, I. J., Yung, J. S. & Singleton, M. R. Biophysical characterization of the centromere-specific nucleosome from budding yeast. *J Biol Chem* **286**, 4021-4026 (2011).
6. Lesbats, P. *et al.* Structural basis for spumavirus GAG tethering to chromatin. *Proc. Natl. Acad. Sci. USA* **114**, 5509-5514 (2017).
7. Minamino, M., Higashi, T. L., Bouchoux, C. & Uhlmann, F. Topological in vitro loading of the budding yeast cohesin ring onto DNA. *Life Sci Alliance* **1** (2018).
8. Wittmeyer, J., Saha, A. & Cairns, B. DNA translocation and nucleosome remodeling assays by the RSC chromatin remodeling complex. *Methods Enzymol* **377**, 322-343 (2004).
9. Munoz, S., Passarelli, F. & Uhlmann, F. Conserved roles of chromatin remodellers in cohesin loading onto chromatin. *Curr Genet* **66**, 951-956 (2020).
