# Supplementary material for: Functional crosstalk between the cohesin loader and chromatin remodelers
Source: Nat Commun. 2022 Dec 13;13:7698. doi: 10.1038/s41467-022-35444-6 (PMC9744909; doi:10.1038/s41467-022-35444-6)
Supplement: Supplementary file 3 — Description of additional Supplementary File [file 41467_2022_35444_MOESM3_ESM.pdf]

Description of additional supplementary files

Supplementary Dataset 1: Peptide crosslinks detected in the CLMS experiment.
