# Supplementary material for: Functional crosstalk between the cohesin loader and chromatin remodelers
Source: Nat Commun. 2022 Dec 13;13:7698. doi: 10.1038/s41467-022-35444-6 (PMC9744909; doi:10.1038/s41467-022-35444-6)
Supplement: Supplementary file 5 — Reporting Summary [file 41467_2022_35444_MOESM5_ESM.pdf]

nature portfolio

Corresponding author(s): Sofía Muñoz (sofiamf@usal.es)
Frank Uhlmann (frank.uhlmann@crick.ac.uk)

Last updated by author(s): Nov 26, 2022

Reporting Summary

Nature Portfolio wishes to improve the reproducibility of the work that we publish. This form provides structure for consistency and transparency in reporting. For further information on Nature Portfolio policies, see our Editorial Policies and the Editorial Policy Checklist.

Statistics

For all statistical analyses, confirm that the following items are present in the figure legend, table legend, main text, or Methods section.

n/a	Confirmed	
☐	☒	The exact sample size (*n*) for each experimental group/condition, given as a discrete number and unit of measurement
☐	☒	A statement on whether measurements were taken from distinct samples or whether the same sample was measured repeatedly
☐	☒	The statistical test(s) used AND whether they are one- or two-sided<br>*Only common tests should be described solely by name; describe more complex techniques in the Methods section.*
☒	☐	A description of all covariates tested
☐	☒	A description of any assumptions or corrections, such as tests of normality and adjustment for multiple comparisons
☐	☒	A full description of the statistical parameters including central tendency (e.g. means) or other basic estimates (e.g. regression coefficient) AND variation (e.g. standard deviation) or associated estimates of uncertainty (e.g. confidence intervals)
☐	☒	For null hypothesis testing, the test statistic (e.g. *F*, *t*, *r*) with confidence intervals, effect sizes, degrees of freedom and *P* value noted<br>*Give P values as exact values whenever suitable.*
☒	☐	For Bayesian analysis, information on the choice of priors and Markov chain Monte Carlo settings
☒	☐	For hierarchical and complex designs, identification of the appropriate level for tests and full reporting of outcomes
☒	☐	Estimates of effect sizes (e.g. Cohen's *d*, Pearson's *r*), indicating how they were calculated

Our web collection on statistics for biologists contains articles on many of the points above.

Software and code

Policy information about availability of computer code

Data collection	MaxQuant (v.2.1.4.0) was used for mass spectrometry data collection. Unicorn (v7.6.0.1306) was used for protein purification using an Äkta Purifier. Amersham 600 (v1.2.0) software was used for immunoblot image acquisition and Typhoon FLA 9500 (v1.1) control software for the acquisition of ATPase assay and Nucleosome sliding assay images.
Data analysis	Protein crosslink mass spectrometry data was analysed using Proteome Discoverer (v2.2, ThermoScientific), XLinkX (v2.2, https://www.hecklab.com/software/xlinkx/) and xiVIEW (https://xiview.org/xiNET_website/index.php). ImageQuant TL (v8.1.0.0) was used for the ATPase assay and Nucleosome sliding assay image quantification, and statistical analyses were performed using GraphPad Prism (v7.0c). MNase-seq data was analysed using cutadapt (v1.9.1), BWA (v0.5.9-r16) and DANPOS2 dpos command (v2.2.2). Clustal (vOmega, https://www.ebi.ac.uk/Tools/msa/clustalo/) was used for amino acid sequence alignment.

For manuscripts utilizing custom algorithms or software that are central to the research but not yet described in published literature, software must be made available to editors and reviewers. We strongly encourage code deposition in a community repository (e.g. GitHub). See the Nature Portfolio guidelines for submitting code & software for further information.

Data

Policy information about availability of data

All manuscripts must include a data availability statement. This statement should provide the following information, where applicable:

- Accession codes, unique identifiers, or web links for publicly available datasets
- A description of any restrictions on data availability
- For clinical datasets or third party data, please ensure that the statement adheres to our policy

The CLMS data generated in this study are contained in Supplementary Dataset 1, the raw data is available at the ProteomeXchange Consortium via the PRIDE partner repository, accession number PXD033446 (http://www.ebi.ac.uk/pride/archive/projects/PXD033446). The MNase sequencing datasets have been deposited with the Gene Expression Omnibus, accession number GSE197657 (https://www.ncbi.nlm.nih.gov/geo/query/acc.cgi?acc=GSE197657). Source data are provided with this paper. The source data, which includes all unprocessed gel images and raw data has also been placed in the Mendeley repository where it can be accessed at https://data.mendeley.com/datasets/vhcrwy6z5f/draft?a=36d56553-aca0-4684-aabb-817a312eed9e.

Human research participants

Policy information about studies involving human research participants and Sex and Gender in Research.

Reporting on sex and gender	n/a
Population characteristics	n/a
Recruitment	n/a
Ethics oversight	n/a

Note that full information on the approval of the study protocol must also be provided in the manuscript.

Field-specific reporting

Please select the one below that is the best fit for your research. If you are not sure, read the appropriate sections before making your selection.

[x] Life sciences [ ] Behavioural & social sciences [ ] Ecological, evolutionary & environmental sciences

For a reference copy of the document with all sections, see nature.com/documents/nr-reporting-summary-flat.pdf

Life sciences study design

All studies must disclose on these points even when the disclosure is negative.

Sample size	This study is based on experiments performed with purified proteins or yeast cultures. Sampling during time-course analyses was at intervals that capture the characteristic kinetics and continued until the reaction containing wild-type components was complete.
Data exclusions	No data was excluded.
Replication	At least three biological replicates of each experiment were performed. In case of quantitative readouts, the quantification of all three experiments is shown, next to the primary data of one of the repeat experiments. In case of qualitative readouts (co-immunoprecipitation experiments), the repeat experiments in all cases confirmed reproducibility and one representative example is shown.
Randomization	No randomization was used or applicable in this research.
Blinding	Blinding was not applicable in this study, as data acquisition and quantification was performed by machines.

Reporting for specific materials, systems and methods

We require information from authors about some types of materials, experimental systems and methods used in many studies. Here, indicate whether each material, system or method listed is relevant to your study. If you are not sure if a list item applies to your research, read the appropriate section before selecting a response.

Materials & experimental systems

n/a	Involved in the study
☐	☒ Antibodies
☒	☐ Eukaryotic cell lines
☒	☐ Palaeontology and archaeology
☒	☐ Animals and other organisms
☒	☐ Clinical data
☒	☐ Dual use research of concern

Methods

n/a	Involved in the study
☒	☐ ChIP-seq
☒	☐ Flow cytometry
☒	☐ MRI-based neuroimaging

Antibodies

Antibodies used

Anti-HA-Tag (clone F-7): Santa Cruz Biotechnology (sc-7392)
Anti-V5(Pk)-Tag (clone SV5-Pk1): BioRad (MCA1360)
Anti-HA-Tag, HRP conjugated (clone GG8-1F3.3.1): Miltenyi Biotec (130-091-972)
Anti-Auxin Inducible Degron (AID/IAA17)-Tag: 2B Scientific (CAC-APC004AM).
Anti-Sth1 (a gift from B. Cairns; Cairns, B. R. et al. RSC, an essential, abundant chromatin-remodeling complex. Cell 87, 1249-1260 (1996))

Validation

Epitope tag antibodies were validated by the respective manufacturers.
Anti-HA-Tag: https://datasheets.scbt.com/sc-7392.pdf
Anti-V5(Pk)-Tag: https://www.bio-rad-antibodies.com/static/datasheets/mca13/viral-v5-tag-antibody-sv5-pk1-mca1360.pdf
Anti-HA-Tag, HRP conjugated: https://www.miltenyibiotec.com/GB-en/products/ha-antibody-gg8-1f3-3-1.html#gref
Anti-AID-Tag: https://www.2bscientific.com/Products/Cosmo-Bio-Ltd/CAC-APC004AM/Anti-AID-Tag-IAA17-Protein

Additional validation of all epitope tag antibodies was performed in our laboratory using cell extracts from cells lacking the respective epitope tags. In all cases, this resulted in loss of the detected band, confirming that the antibodies specifically recognize their epitope-tagged targets.

The Anti-Sth1 antibody was described and validated in: Cairns, B. R. et al. RSC, an essential, abundant chromatin-remodeling complex. Cell 87, 1249-1260 (1996). Additionally, we confirmed that the antibody recognizes the Sth1 subunit in the purified RSC complex, but not any of the other RSC subunits, nor any of the other proteins present in our interaction analyses.
